# A cross sectional study on fertility knowledge in Japan, measured with the Japanese version of Cardiff Fertility Knowledge Scale (CFKS-J)

**DOI:** 10.1186/1742-4755-12-10

**Published:** 2015-01-31

**Authors:** Eri Maeda, Hiroki Sugimori, Fumiaki Nakamura, Yasuki Kobayashi, Joseph Green, Machi Suka, Masako Okamoto, Jacky Boivin, Hidekazu Saito

**Affiliations:** Graduate School of Medicine, The University of Tokyo, 7-3-1, Hongo, 113-0033 Bunkyo-ku, Tokyo, Japan; Department of Preventive Medicine, Graduate School of Sports and Health Sciences, Daito Bunka University, 560, Iwadono, 355-8501 Higashimatsuyama-shi, Saitama, Japan; Department of Public Health and Environmental Medicine, The Jikei University School of Medicine, 3-25-8, Nishishinba-shi, 105-8461 Minato-ku, Tokyo, Japan; Department of Applied Biological Chemistry, Graduate School of Agricultural and Life Sciences, The University of Tokyo, 7-3-1, Hongo, 113-0033 Bunkyo-ku, Tokyo, Japan; Cardiff Fertility Studies Research Group, School of Psychology, Cardiff University, Tower Building, Park Place, CF10 3AT Cardiff, South Wales, UK; Center of Maternal-Fetal, Neonatal and Reproductive Medicine, National Center for Child Health and Development, 2-10-1, Okura, 157-0074 Setagaya-ku Tokyo, Japan; Research Fellow of Japan Society for the Promotion of Science, Tokyo, Japan

**Keywords:** Fertility awareness, Knowledge, Education, Cardiff fertility knowledge scale, Japan

## Abstract

**Background:**

A recent survey of 79 countries showed that fertility knowledge was lower in Japan than in any other developed country. Given the fertility decline in Japan and the importance of fertility knowledge, we conducted an online survey to examine fertility knowledge and the related factors for effective public education.

**Methods:**

We studied people aged 18-59 years old, n = 4,328 (the “General” group), and also people who had been trying to conceive for at least six months, 18-50 years old, n = 618 (the “Triers” group). Fertility knowledge was assessed using the Japanese version of the 13-item Cardiff Fertility Knowledge Scale (CFKS-J). All participants provided socio-demographic and fertility information. Participants also completed a 14-item health literacy scale and an 11-item health numeracy scale. We asked participants who were aware of age-related decline in fertility when and where they first acquired that knowledge.

**Results:**

The average percentages of CFKS-J items answered correctly were 53.1% in the Triers group and 44.4% in the General group (p < 0.001). Multivariate linear regression models showed in the Triers group greater fertility knowledge was associated with greater health literacy and prior medical consultation regarding their fertility. In the General group greater fertility knowledge was associated with being female, younger, university educated, currently trying to conceive, non-smoking, having higher household income, having higher health literacy and having higher health numeracy. Of those who were aware of the age-related decline in fertility, around 3% first learned the fact “at school”, and around 65% first learned it “through mass media” or “via the Internet”. More than 30% of the respondents first learned it “less than 5 years before” the survey.

**Conclusions:**

Although fertility knowledge had improved since a previous study, possibly due to recent media coverage of age-related infertility, it was still low. Educational interventions, both in schools and in the community, may be needed to increase fertility knowledge in the general population because most people obtain fertility knowledge from mass media, which has been shown to often present distorted and inaccurate fertility information.

## Background

The trend to delay childbearing and the decline in fertility are serious concerns regarding reproductive-health in developed countries [1, 2]. In addition to pursuit of career goals and diverse lifestyles, a lack of accurate information on which to base informed fertility decisions could account in part for this trend. Misinformation about fertility could explain sub-optimal fertility behaviour, and public education campaigns should be considered as ways of increasing fertility knowledge [3–5]. Indeed, fertility knowledge in many populations is poor [6–11]. Government-sponsored educational initiatives have been undertaken in some countries (e.g., Belgium) [12], but more needs to be known about the factors that predict fertility knowledge so these factors can be addressed in educational interventions.

In Japan, the total fertility rate has been decreasing (1.41 in 2012) and the parental age at first birth has been increasing (30.3 and 32.3 years for women and men, respectively in 2012) [13]. These trends continue despite concerted government effort to introduce policies that address work-life balance and deficits in childcare [14]. Recently, it has been recognised that many Japanese people lack fertility knowledge and that this lack of knowledge could also play a role in fertility trends in Japan. One surprising finding of a recent survey was that 36.4% of young women estimated their own age limit for natural pregnancy to be between 45 and 60 years [15]. Furthermore, an international survey of 79 countries (the International Fertility Decision-making Study, IFDMS) [16] showed that fertility knowledge was lower in Japan than in any other developed country. This was quite a contrast to scholastic ability [17] and to health numeracy [18], both of which were very high among Japanese people. This severe lack of fertility knowledge can be attributed to social taboos against referring to sex or female age, and also to the fact that sex education in school is generally focused on prevention of pregnancy and sexually transmitted infections (STIs) [19]. To remedy this situation, the government proposed the need for fertility education in 2013 [20]. The proposal sparked much public debate, which was covered by mass media intensively [21].

The aims of the present study were to examine fertility knowledge in the general population and to investigate the related factors, which will provide important background for effective public education. We investigated knowledge in two groups: a representative sample of the general population (‘General’ group) and a sample consisting of people who were currently trying to conceive (‘Triers’ group). The Triers group was studied to replicate the IFDMS research in Japan. The General group was recruited to represent people who would be the target group in future public-education campaigns. We included future, current, and post-reproductive generations in the target population because older people generally transmit information, whether it is correct or incorrect, to younger generations. In line with the previous studies examining factors associated with fertility knowledge [7, 16, 22–24], it was hypothesized that fertility knowledge would be greater in women, in people of higher socio-economic status (SES), and in those with more interest in childbearing: those currently trying to conceive and those with a history of medical consultation for infertility. According to previous studies, health awareness and health-related activities such as tobacco smoking [24] are associated with fertility knowledge. It was also hypothesized that greater fertility knowledge would be found in people who had greater skills regarding their health, such as those in non-smoking status and those with higher health literacy and numeracy.

## Methods

### Participants

Participants were recruited via online social research panels (SRPs). Inclusion criteria for the General group were that respondents were men and women aged between 18 and 59 years old (n = 4,328). The inclusion criteria for the Triers group were that respondents were men and women aged between 18 and 50, currently married or living with their partner, currently trying to conceive (Triers) for at least 6 months, and not pregnant (n = 618). The criteria for the Triers group were the same as those used in the IFDMS [16].

### Procedures

**Figure 1:**
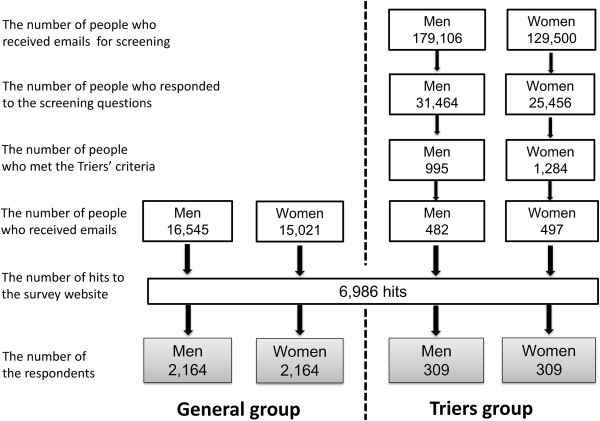
**Flowchart showing the procedure.**

An online market research company (Macromil, Tokyo, Japan), which has a nationwide research panel (SRP) of more than 1 million registrants, sent recruitment emails to people who were randomly selected from its registrants (Figure 1). Recruitment e-mails for the General group were sent to 31,566 eligible people aged 18 – 59 years. To recruit the participants for the Triers group, 308,606 people aged 18 – 50 years were pre-screened and 979 eligible people received the recruitment emails. We performed quota-sampling equally by gender and age-group blocks (i.e., men and women in 18 – 29, 30 – 39, 40 – 49, and 50 – 59 year-old age groups). Recruitment continued until the intended number of participants in each block had been recruited. Medical professionals and advertising professionals were excluded during the recruitment. Participants were provided a reward incentive consistent with the SRPs procedure. All the procedures were completed from September 30 to October 9, 2013.

### Questionnaire design

This survey consisted of 117 items covering 8 domains, which were developed to investigate the factors associated with low fertility in Japan. Only those questions relevant to analyses presented in this paper are described here. To ensure that the questionnaire was understandable, we conducted two pilot surveys on a small group of our colleagues and about 100 people registered with the above mentioned panel.

#### Fertility knowledge

The Japanese version of the Cardiff Fertility Knowledge Scale (CFKS) [16] (CFKS-J) was developed using forward translation and back-translation of the original CFKS. Forward translation was conducted by two Japanese public health researchers (EM and FN). A bilingual Japanese-English individual translated the resulting questionnaire back into English, and a native English speaker evaluated the concordance between the back-translated items and the originals. After concordance was confirmed, minor changes were made, based on discussion between the Japanese researchers (EM and FN) and the developer of the original CFKS in Cardiff University (JB).

The CFKS consists of 13 items that measure knowledge about fertility facts, risks, and myths. All items were rated on a three point scale of ‘true’, ‘false’, or ‘do not know’. A correct answer was assigned one point and an incorrect or ‘do not know’ answer was assigned zero points. Scores are reported as percents of the highest possible score. Internal consistency coefficient alpha of the original CFKS was 0.79, and an exploratory factor analysis showed that all items loaded >0.30 on one general factor that accounted for 30% of between-item variance [16].

#### Source of information

The first item of the CFKS-J asks whether the statement “A woman is less fertile after the age of 36 years” is true or false. We asked participants who answered that item correctly when and where (e.g. at school, through mass media) they first acquired that knowledge. We asked them when and where they first learned about prevention of pregnancy and STIs as well.

#### Socio-demographic variables

Gender, age in years, and categorized annual household income of the participants were provided by the online market research company. Annual household income was categorized into 4 groups: low, < 4 million Japanese Yen (JPY); moderate, 4 – 7 million JPY; high, ≥ 8 million JPY; and “unknown”. At the time of the study 1 Japanese Yen = 0.0076 Euro. Participants also indicated their academic background (university education, yes/no) and marital status (married, yes/no).

#### Fertility status

Participants reported whether they had given birth or fathered a child. Participants in the General group indicated whether they were currently trying to conceive (yes/no) and whether they hoped to have children in the future (yes/no). Participants in the Triers group stated the number of months they had been trying to conceive, and whether they had sought a medical consultation or treatment regarding fertility (yes/no).

#### Health-related skills and behaviour

We used the 14-item health literacy scale (HLS-14) for measuring health literacy [25]. The HLS-14 measures functional (5 items, e.g., “The print is too small for me when I read instructions or leaflets from hospitals or pharmacies”), communicative (5 items, e.g., “I collect information from various sources if I am diagnosed as having a disease”), and critical health literacy (4 items, e.g., “I consider whether the information is credible if I am diagnosed as having a disease”). Each item has 5 response choices. The total score is the sum of the scores on each item, and higher scores indicate better health literacy.

Health numeracy was measured using the Japanese version of the Lipkus scale (the Lipkus-J) [18, 26]. The Lipkus-J is a uni-dimensional 11-item scale that focuses on the basic understanding of math and probability relevant to health risks. Item responses were dichotomized to be either correct or incorrect. The total score was calculated as the number of correct items for each respondent. The range was 0 to 11 with higher scores indicating better numeracy.

Additionally, we asked participants about tobacco smoking, as an indicator of health behaviour (smoke more than a few times a week: yes/no).

### Data analyses

Internal consistency coefficient alpha, bi-serial item correlations, and tetrachoric correlations among items were used to evaluate the CFKS-J. A factor analysis was conducted using the matrix of tetrachoric correlations. Factors with loadings ≥ 0.3 were retained.

Descriptive statistics were used to describe performance on CFKS-J and the named sources of information. T-tests and analysis of variance (ANOVA) were used to compare the total scores on the CFKS-J between socio-demographic categories and between fertility-status categories. Pearson’s correlation was used to examine the association between age in years and CFKS-J score. Spearman’s correlation was used to explore the associations among scores on the HLS-14, the Lipkus-J, and the CFKS-J. The *t*-test for independent samples was used to compare results from the present study with results from the IFDMS [16].

Multivariate linear regression using the robust estimator of variance was used to assess the relation between the total score on the CFKS-J and the variables described above. All the analyses were performed using STATA12-SE (StataCorp LP, College Station, TX, USA).

### Ethics statement

Ethical review and approval was carried out at the institutional ethics committee of the National Center for Child Health and Development (Heisei 25 nendo-10), Daito Bunka University (July 30, 2013), and the University of Tokyo (10346). Collection of online data complied with the “Code of Conduct of Marketing Research” and “Requirements for Compliance Program on Personal Information Protection (JIS Q 15001)” of the Japan Marketing Research Association.

## Results

### Study population

Table 1 shows the characteristics of the General and Trier groups. The proportion of respondents in each gender and age group was controlled during recruitment to make them similar to the proportions in the general population of Japan.

**Table 1:** **Means (standard deviations) or frequencies (n, %) of variables in the study samples**

	General group (n = 4,328)	Triers group (n = 618)
**Socio-demographic**		
Gender (n, %)		
Male	2,164 (50.0)	309 (50.0)
Female	2,164 (50.0)	309 (50.0)
Age in years (M, SD)	39.3 (11.2)	35.2 (6.9)
University education (n, % yes)	1,854 (42.8)	290 (46.9)
Annual household income (n, %)		
<4 million JPY	1,188 (27.4)	176 (28.5)
4 - 7 million JPY	1,515 (35.0)	269 (43.5)
≥8 million JPY	701 (16.2)	76 (12.3)
Unknown	924 (21.3)	97 (15.7)
Ever married (n, %)	2,722 (62.9)	-
**Fertility status**		
Given birth/fathered child (n, % yes)	2,143 (49.5)	242 (39.2)
Currently trying to conceive (n, % yes)	312 (7.2)	-
Hope to have children in the future (n, % yes)	1,731 (40.0)	-
Prior medical consultation for fertility (n, % yes)	-	215 (34.8)
Number of years trying to conceive (M, SD)	-	2.9 (5.4)
**Health-related skills and behaviour**		
Smoking (n, %, yes)	817 (18.9)	128 (20.7)
The score of HLS-14 (M, SD)	49.6 (7.2)	50.8 (7.4)
The score of the Lipkus-J (M, SD)	9.18 (2.2)	9.17 (2.0)

Note. Range for Health Literacy Scale (HLS)-14 = 14 - 70; Lipkus-J = 0 - 11.

### Reliability testing and validation testing of the CFKS-J

The CFKS-J scores were normally distributed in the General and Triers groups. Internal consistency reliability was moderate: coefficient alpha = 0.74 (General group), 0.72 (Triers group). A single to 3-factor solution was computed to examine the dimensionality of the 13 items. In the General group, the eigenvalues of the factors were 4.14, 1.33, and 0.56, which accounted for 72%, 23%, and 10% of the variance, respectively. The high eigenvalue of the second factor was attributed to a high tetrachoric correlation between item 3 (“Smoking decreases female fertility”) and item 4 (“Smoking decreases male fertility”) (*r*_*tetrachoric*_ = 0.89), because the eigenvalues of the factors changed to 3.71, 0.88, 0.35 when item 3 was dropped from the analysis. The biserial item correlations with the underlying factor ranged from 0.36 to 0.55 and the factor loadings for the first factor ranged from 0.30 to 0.67. The results in the Triers group were similar (Table 2). Overall, the CFKS-J appeared to have a one-factor structure, as did the original CFKS.

**Table 2:** **Factor loadings for each item of CFKS-J in the General (n = 4,328) and Triers (n = 618) group**

		Factor loading ^a^	
	Item	General group	Triers group
1	A woman is less fertile after the age of 36 years. [True]	0.62	0.58
2	A couple would be classified as infertile if they did not achieve a pregnancy after one year of regular sexual intercourse without using contraception. [True]	0.40	0.32
3	Smoking decreases female fertility. [True]	0.63	0.63
4	Smoking decreases male fertility. [True]	0.67	0.66
5	About 1 in 10 couples are infertile. [True]	0.56	0.63
6	If a man produces sperm he is fertile. [False]	0.53	0.59
7	These days a woman in her 40s has a similar chance of getting pregnant as a woman in her 30s. [False]	0.59	0.67
8	Having a healthy lifestyle makes you fertile. [False]	0.65	0.72
9	If a man has had mumps after puberty he is more likely to later have a fertility problem. [True]	0.44	0.38
10	A woman who never menstruates is still fertile. [False]	0.30	0.34
11	If a woman is overweight by more than 13 kg then she may not be able to get pregnant. [True]	0.54	0.51
12	If a man can achieve an erection then it is an indication that he is fertile. [False]	0.66	0.66
13	People who have had a sexually transmitted disease are likely to have reduced fertility. [True]	0.60	0.55

^a^Only factor loadings for the first factor shown.

### Performance on the fertility knowledge scale

**Figure 2:**
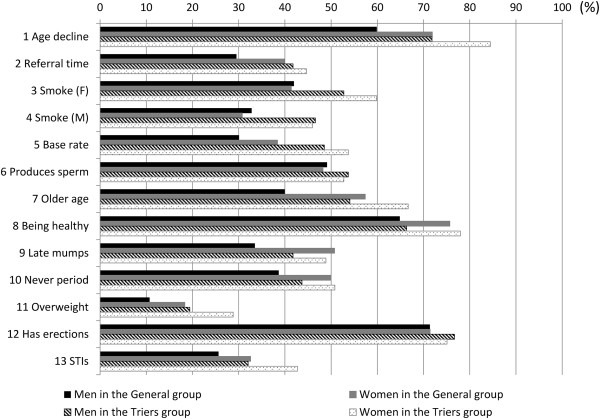
**The percentage of participants who answered correctly to each item of CFKS-J.**

The average percent correct scores on the CFKS-J were 53.1 (SD = 23.4) in the Triers group and 44.4 (SD = 23.1) in the General group (t = -8.77, p < 0.001). Figure 2 shows the percentage of participants who answered each item correctly, by group and gender.

Univariate relations between the total score of CFKS-J and study variables are shown in Table 3 (the General group) and Table 4 (the Triers group). With regards to annual household incomes, 924 people in the General group and 97 people in the Triers group who answered “unknown” were excluded from the analysis. As shown in Table 3 for the General group CFKS-J scores were significantly higher in those who were female, university educated, married, currently trying to conceive, non-smoking, had higher household income, or had given birth or fathered a child. There were low positive correlations of the CFKS-J with the HLS-14 (ρ = 0.24, p < 0.001) and with the Lipkus-J (ρ = 0.10, p < 0.001). In the multivariate linear regression analysis, we excluded marital status from the model in the General group due to its multi-collinearity with whether participants had given birth or fathered a child. In the General group (n = 3,404) the factors that remained independent predictors of greater fertility knowledge in the multivariate model were being female, younger, university educated, currently trying to conceive, non-smoking, having higher household income, having higher score on the HLS-14, and having higher score on the Lipkus-J (R^2^ for the model = 0.095) (Table 3).

**Table 3:** **Univariate and multivariate analyses for factors related to fertility knowledge in the General group**

	Univariate association (n = 4,328)		Multivariate linear regression (n = 3,404, R-squared for model = 0.095)		
Variables	Mean (SD)	P	Coefficient ^a^	95% confidence interval	P
**Socio-demographic**					
Gender					
Male	40.6 (23.6)		Reference		
Female	48.2 (21.9)	<0.001	6.54	4.95 to 8.15	<0.001
Age					
Each additional year older	-0.02^b^	0.16	-0.13	-0.21 to -0.06	<0.001
Annual household income					
< 4 million JPY	43.2 (22.4)		Reference		
4 - 7 million JPY	45.9 (22.9)		1.43	-0.28 to 3.15	0.10
≥ 8 million JPY	47.0 (23.8)	<0.001	2.57	0.34 to 4.79	0.024
Educational level					
No university education	42.4 (22.8)		Reference		
University education	47.1 (23.2)	<0.001	3.33	1.73 to 4.93	<0.001
Marital status					
Never married	41.7 (23.2)				
Ever married	46.0 (22.8)	<0.001			
**Fertility**					
Whether or not given birth					
Not given birth/fathered a child	43.2 (23.8)		Reference		
Given birth/fathered a child	45.7 (22.3)	<0.001	0.84	-0.79 to 2.47	0.31
Whether or not currently trying to conceive					
Not currently trying to conceive	43.6 (22.9)		Reference		
Currently trying to conceive	54.7 (22.8)	<0.001	6.54	3.62 to 9.46	<0.001
**Health-related skills and behaviour**					
Smoking status					
Non-smoking	45.4 (23.2)		Reference		
Smoking	40.2 (22.2)	<0.001	-2.29	-4.12 to -0.45	0.015
The score of HLS-14					
each additional score higher	0.24^c^	<0.001	0.52	0.41 to 0.63	<0.001
The score of the Lipkus-J					
each additional score higher	0.10^c^	<0.001	0.90	0.54 to 1.27	<0.001

Note: With regards to annual household incomes, 924 people in the General group who answered “unknown” were excluded from the analysis.

^a^Non-standardized coefficient. ^b^Pearson’s correlation coefficient.

^c^Spearman’s correlation coefficient.

**Table 4:** **Univariate and multivariate analyses for factors related to fertility knowledge in the Triers group**

	Univariate association (n = 618)		Multivariate linear regression (n = 521, R-squared for model = 0.13)		
Variables	Mean (SD)	P	Coefficient ^a^	95% confidence interval	P
**Socio-demographic**					
Gender					
Male	49.9 (23.8)		Reference		
Female	56.3 (22.7)	<0.001	3.28	-0.86 to 7.41	0.12
Age					
each additional year older	-0.01^b^	0.72	-0.22	-0.52 to 0.08	0.16
Annual household income					
< 4 million JPY	50.9 (24.1)		Reference		
4 - 7 million JPY	53.0 (23.8)		1.56	-2.95 to 6.07	0.50
≥ 8 million JPY	53.0 (23.5)	0.62	1.03	-5.24 to 7.30	0.75
Educational level					
No university education	53.5 (23.5)		Reference		
University education	52.7 (23.3)	0.69	-0.94	-5.39 to 3.51	0.68
**Fertility**					
Whether or not given birth					
Not given birth/fathered a child	54.6 (23.6)		Reference		
Given birth/fathered a child	50.8 (23.0)	0.046	-3.38	-7.42 to 0.67	0.10
How long trying to conceive					
Trying to conceive < 12 months	50.5 (23.2)		Reference		
Trying to conceive ≥ 12 months	54.2 (23.5)	0.08	0.15	-4.71 to 5.02	0.95
Prior medical consultation					
Not consulted a medical doctor	48.6 (23.2)		Reference		
Consulted a medical doctor	61.6 (21.5)	<0.001	11.64	7.28 to 16.0	<0.001
**Health-related skills and behaviour**					
Smoking status					
Non-smoking	54.3 (22.8)		Reference		
Smoking	48.7 (25.3)	0.02	-1.89	-7.24 to 3.46	0.49
The score of HLS-14					
Each additional score higher	0.22^c^	<0.001	0.59	0.30 to 0.88	<0.001
The score of the Lipkus-J					
Each additional score higher	0.072^c^	0.08	0.27	-0.80 to 1.35	0.62

Note: With regards to annual household incomes, 97 people who answered “unknown” were excluded from the analysis.

^a^Non-standardized coefficient. ^b^Pearson’s correlation coefficient.

^c^Spearman’s correlation coefficient.

As shown in Table 4 for the Triers group, the CFKS-J score was significantly higher in those who were female, non-smoking, had not given birth or fathered a child, or had prior medical consultation regarding their fertility. There was a low positive correlation between the CFKS-J and the HLS-14 (ρ = 0.22, p < 0.001). In the Triers group the factors that remained independent predictors of greater fertility knowledge in the multivariate model were having a higher score on the HLS-14 and having a prior medical consultation regarding fertility (R^2^ for the model = 0.13) (Table 4).

### Source of information

A total of 3,334 participants (2,851 in the General group and 483 in the Triers group) answered the question about the age-related decline in fertility correctly (Table 5). Of those 3,334, around 3% first learned that fact about fertility “at school” and around 65% first learned it “through mass media” or “via the Internet”. In contrast, more than 30% first learned about prevention of pregnancy and STIs “at school”. With the two groups combined, 33% of the respondents first learned that fact about fertility “less than 5 years before” the survey, whereas around 80% first learned about prevention of pregnancy and STIs “more than 10 years before” the survey.

**Table 5:** **Sources of information about fertility, contraception and STIs in people who knew about age-related infertility**

	Fertility (n, %)			Contraception (n, %)			STIs (n, %)		
	Total (n = 3,334)	General group (n = 2,851)	Triers group (n = 483)	Total (n = 3,334)	General group (n = 2,851)	Triers group (n = 483)	Total (n = 3,334)	General group (n = 2,851)	Triers group (n = 483)
**Where first learned**									
At school	102 (3.1)	89 (3.1)	13 (2.7)	1147 (34.4)	967 (33.9)	180 (37.3)	1060 (31.8)	889 (31.2)	171 (35.4)
At medical institutions	213 (6.4)	152 (5.3)	61 (12.6)	25 (0.8)	16 (0.6)	9 (1.9)	139 (4.2)	110 (3.9)	29 (6.0)
From family members	30 (0.9)	26 (0.9)	4 (0.8)	38 (1.1)	30 (1.1)	8 (1.7)	20 (0.6)	18 (0.6)	2 (0.4)
From partners	89 (2.7)	61 (2.1)	28 (5.8)	84 (2.5)	65 (2.4)	19 (3.9)	42 (1.3)	35 (1.2)	7 (1.5)
From friends	72 (2.2)	60 (2.1)	12 (2.5)	526 (15.8)	457 (16.0)	69 (14.3)	216 (6.5)	186 (6.5)	30 (6.2)
Through mass media	1828 (54.8)	1587 (55.6)	241 (50.0)	560 (16.8)	483 (16.9)	77 (15.9)	936 (28.1)	812 (28.5)	124 (25.7)
Via the Internet	354 (10.6)	278 (9.8)	76 (15.7)	72 (2.2)	59 (2.1)	13 (2.7)	186 (5.6)	156 (5.5)	30 (6.2)
Don’t know	646 (19.4)	598 (21.0)	48 (9.9)	882 (26.5)	774 (27.2)	108 (22.4)	735 (22.0)	645 (22.6)	90 (18.6)
**When first learned**									
<6 months before	91 (2.7)	84 (3.0)	7 (1.5)	10 (0.3)	10 (0.4)	0 (0)	6 (0.2)	6 (0.2)	0 (0)
6 months - 1 year before	125 (3.7)	105 (3.7)	20 (4.1)	7 (0.2)	5 (0.2)	2 (0.4)	11 (0.3)	9 (0.3)	2 (0.4)
1 - 3 years before	421 (12.6)	330 (11.6)	91 (18.9)	23 (0.7)	17 (0.6)	6 (1.2)	32 (1.0)	27 (1.0)	5 (1.0)
3 - 5 years before	485 (14.5)	368 (13.0)	117 (24.2)	58 (1.7)	51 (1.8)	7 (1.5)	84 (2.5)	69 (2.4)	15 (3.1)
5 - 10 years before	628 (18.8)	519 (18.2)	109 (22.6)	186 (5.6)	163 (5.7)	23 (4.8)	268 (8.0)	220 (7.7)	48 (10.0)
≥10 years before	914 (27.4)	831 (29.2)	83 (17.2)	2757 (82.7)	2346 (82.3)	411 (85.1)	2547 (76.4)	2179 (76.4)	368 (76.2)
Don’t know	670 (20.1)	614 (21.5)	56 (11.6)	293 (8.8)	259 (9.1)	34 (7.0)	386 (11.6)	341 (12.0)	45 (9.3)

## Discussion

The main findings of this study confirm that fertility knowledge in Japan is low and that knowledge varies with literacy issues and experience with trying to conceive. Further, the findings show that people mainly recall acquiring their knowledge from non-formal sources such as media and the Internet. More formal sources of education need to be considered if fertility health is to be improved.

A strength of the study was the use of the validated CFKS (fertility risk factors, misconceptions and basic facts) [16]. To date, CFKS is the sole validated fertility knowledge scale although there are many other scales which address broader knowledge including misconceptions about infertility treatments [4–8]. In addition, the large sample size allowed statistical adjustment for likely covariates (i.e., household income and educational level), and the inclusion of men and of people beyond their reproductive years also gives new information, as previous studies mainly focused on women [4, 6, 7, 27, 28] or students [4, 5, 10, 23, 29, 30].

Our findings are consistent with those of previous studies that investigated the relationship between fertility knowledge and background factors. The IFDMS reported that greater fertility knowledge was associated with female gender, university education, paid employment, very high Human Development Index, and prior medical consultation for infertility [16]. Other previous studies showed higher knowledge in women, those with higher education [7, 22] and non-smokers [24] and lower knowledge in men and in those of lower SES [23]. In the present study we replicated those findings: fertility knowledge was significantly higher among women, those of higher SES (i.e. being university educated or having higher household income), those who had experience with trying to conceive (i.e. currently trying to conceive or having prior medical consultation), and those who had better health-related skills and behaviour (i.e. non-smokers, having higher health literacy or numeracy).

Interest in childbearing was strongly associated with fertility knowledge: In the General group, people who were currently trying to conceive had scores that were 6.5 percentage points higher than those who were not currently trying to conceive, and in the Triers group those with a history of medical consultation or treatment for fertility had scores that were 11.6 percentage points higher than the others. However, to prevent infertility due to a lack of fertility knowledge, people without current interest in childbearing need to know the facts while they have time to protect their own health and to make choices about childbearing. We should also be aware of the fertility knowledge of older people. If they have misperceptions or biases regarding fertility, those could be transmitted to younger people. The older people in this study were relatively young when they had first become parents, but their levels of fertility knowledge were low.

Although referring to female age and fertility was once taboo in Japan, the issue has recently been taken up by the media since the national television channel NHK aired a documentary program discussing age-related infertility in 2012 [31]. Subsequently in 2013, the government proposed to create and distribute fertility educational materials [20] and to revise the current subsidy system for assisted reproductive technology treatments, setting the age limit up to 42 years old [32, 33]. These governmental movements sparked much public debate about the need to educate people, and they were covered by mass media intensively [21, 34]. There was public disapproval of the government, as it appeared to force the timing of childbearing on women, but the proposal was supported by some who had so far been given only little information about fertility [21]. The effects of this media coverage may be seen in the present results. Although our results replicate the IFDMS finding, showing that fertility knowledge in Japan was still lower than the average score in developed countries, the average performance in the Triers group, which was selected using the same inclusion criteria as the IFDMS sample, was significantly better (53.1% correct) than the 34% correct that was reported in the IFDMS survey in Japan held in 2009 – 2010 (p < 0.001, [16]). In particular we note that there was a remarkably higher percentage of respondents who correctly answered the question about the age-related decline in female fertility: 78% in the present survey versus 29% in the earlier IFDMS [data on file, Merck Serono]. Considering the fact that 65% of participants learned their fertility knowledge through the mass media or via the Internet, this difference could be due to recent governmental movements to prevent age-related infertility and the intense media coverage that subsequently ensued [21, 34, 35], but because the present study was cross-sectional we cannot be sure that this difference indicates an actual improvement over time. On the other hand, mass media and the Internet often present information that is incomplete, distorted and inaccurate [36]. In the present results, the percentages of participants who correctly answered the questions about smoking and overweight were still low and had not improved, possibly reflecting the fact that mass-media reports of the impact of smoking and of overweight on fertility are very rare.

Some countries are conducting educational interventions to spread accurate information [8, 9, 12]. In view of our findings, the educational interventions should target schools and the community to be the most effective. School education, especially primary education, can target the knowledge of those who showed significantly lower knowledge scores: those who currently do not have the intention of childbearing, those who are not university educated, and those who do not have high health literacy. In general, school-based education is more likely to improve knowledge of those children who belong to a family with higher SES, resulting in widening inequalities of the knowledge on fertility as well as health between higher and lower SES groups, but a previous study regarding smoking prevention interventions showed that social network approaches and community education in addition to school education were effective among low-SES adolescents [37]. Further, schools could provide a more consistent approach to the content than would information provided in the media. This study provides policy-makers with important background for improving the school-based and community educational initiatives that are being undertaken in developed countries.

There are several limitations to be noted. First, this was a cross-sectional survey and thus we cannot infer causality. Second, the use of SRPs could have caused selection bias. Acquiring health-related information via the Internet is associated with higher education levels and higher household incomes [38, 39] and indeed, the percentage of participants who had university education was much higher than that calculated from the 2010 Population Census [40]: 22% in people aged between 20 and 59 years old. Moreover, participants could have looked for answers via the Internet during the survey. Volunteer bias toward those who were more interested in fertility is also possible. Therefore if these results are generalized to the national population then fertility knowledge might be overestimated. However, in that case the agendas we have to address are the same, because the level of fertility knowledge in the general population would be even worse than reported here.

## Conclusions

Although fertility knowledge among people in Japan had improved, possibly due to the recent governmental movements and the media coverage of age-related infertility, it was still lower than the average in developed countries. Given the fact that fertility decisions should be based on correct and complete information, we note that multiple factors are associated with fertility knowledge, especially education and the personal relevance of childbearing. Importantly, many people obtained knowledge through mass media or via the Internet. To prevent infertility and broaden people’s choices about childbearing, educational interventions may be needed both in schools and in the community.

## Abbreviations

ANOVA: Analysis of variance
CFKS: Cardiff Fertility Knowledge Scale
CFKS-J: The Japanese version of the Cardiff Fertility Knowledge Scale
HLS-14: The 14-item health literacy scale
IFDMS: The International Fertility Decision-making Study
JPY: Japanese Yen
Lipkus-J: The Japanese version of the Lipkus scale
SES: Socio-economic status
SRP: Social research panel
STI: Sexually transmitted infection.
